# Coronavirus disease 2019 (COVID-19) outbreak in Iran: Actions and problems

**DOI:** 10.1017/ice.2020.86

**Published:** 2020-03-20

**Authors:** Milad Abdi

**Affiliations:** 1 Student Research Committee, Faculty of Medicine, Iran University of Medical Sciences, Tehran, Iran; 2 Department of Microbiology, Faculty of Medicine, Iran University of Medical Sciences, Tehran, Iran

*To the Editor*—Coronaviruses (CoV) are a large family of viruses that cause illness ranging from the common cold to more severe diseases such as Middle East Respiratory Syndrome (MERS-CoV) and Severe Acute Respiratory Syndrome (SARS-CoV). Investigations have shown that SARS-CoV was transmitted from civet cats to humans and MERS-CoV from dromedary camels to humans. Coronaviruses are present in humans and many different species of animals, including camels, cattle, cats, and bats. Rarely, animal coronaviruses can infect people and then spread between people such as MERS-CoV, SARS-CoV, and the new virus named SARS-CoV-2.^1,2^

The SARS-CoV-2 coronavirus was first detected in China, and the disease it causes has been named “coronavirus disease 2019,” which is abbreviated “COVID-19.” Common signs of this infection include respiratory symptoms, fever, cough, shortness of breath, and breathing difficulties. In more severe cases, infection can cause pneumonia, severe acute respiratory syndrome, kidney failure, and even death.^1,2^

Coronavirus disease (COVID-19) was first reported from Wuhan, China, on December 31, 2019, and is now a concerning issue in the world especially in Iran, South Korea, and Italy.^2,3^ In Iran, on February 19, 2020, two patients in Qom city were confirmed as SARS-CoV-2 positive. Afterward, the disease spread very rapidly in adjacent provinces near Qom, such as Tehran, Markazi, Isfahan, and Semnan provinces, and shortly thereafter in all 31 provinces of the country. By March 8, 2020, according to Dr. Jahanpour, spokesman for the Ministry of Health and Medical Education, the total number of infected people who tested positive for SARS-Cov-2 had reached 6,566 and COVID-19 deaths had reached 194.^4^ Although these data continue to change, they show that the mortality rate has been ∼2.9% in COVID-19–positive cases.^5^ At the time of article submission, Iran ranked third in the number of people suffering from the disease after China and South Korea and second in the number of deaths and recovered cases.^6,7^

According to officials of the Iranian Ministry of Health and Medical Education (MHME), in the coming days the number of positive cases and deaths will increase. Thus, the issue of the COVID-19 outbreak and its control has become a top priority for the MHME. Iran formed the National Committee to Combat Corona and has decided to control this infection using all resources of the country, especially the knowledge, equipment, and skilled personnel. The MHME has initiated the following actions to combat the disease^3,5,8,9^:

(1) Increased awareness and informed people about COVID-19 and recommended protective measures proposed by the World Health Organization (WHO) through media such as television, radio, etc, including the following recommendations:
  - Wash your hands frequently and thoroughly with an alcohol-based hand rub or soap and water.
  - Maintain at least 1 m (3 feet) distance from anyone who is coughing or sneezing.
  - Avoid touching eyes, nose, or mouth with contaminated hands.
  - Practice respiratory hygiene by covering mouth and nose with bent elbow, facial masks, or tissue when you cough or sneeze.
  - Seek medical care early at the onset of fever, cough, and/or difficulty breathing.
(2) Restricted traffic in busy areas such as places of pilgrimage, tourism, and markets.
(3) Closed kindergartens, schools, and universities.
(4) Reduced office working hours.
(5) Cancelled the congregational prayer and Jumu’ah prayer (Friday prayer).
(6) Cancelled all multiplayer sport matches like football and volleyball.
(7) Disinfected busy places such as bus stops, subways, and bus rapid transits (BRTs).
(8) Limited access and identification of people suspected of having COVID-19 at the entrance and exit of a number of cities.
(9) Created groups and teams to diagnose the disease through district health centers located in different areas of the affected cities.

Despite these decisions and actions, many problems remain for Iran in confronting and defeating in the COVID-19 outbreak, including the following^3,9^:

(1) Lack of adequate infrastructure and per capita hospital beds and equipment in some cities.
(2) Inadequate protective equipment such as facial masks, disinfectants, and antiseptics such as alcohol.
(3) Difficulty importing some essential medicines.
(4) Difficulty of strategies such as quarantining cities due to the wide distribution of the virus throughout the country.
(5) Increased risk of virus transmission caused by increased travel due to the New Year holiday (Nowruz, March 19, 2020) and related vacations.

Iran is now fighting COVID-19 with all its might, but the wide spread of the disease in all the provinces of the country has made it extremely difficult to control, and Iran has required assistance from international organizations such as the World Health Organization. The increase in travel related to the Nowruz holiday, which started even 15 days earlier due to the closure of schools and universities, increases the likelihood of transmission and circulation of the virus and increased prevalence of COVID-19. To effectively fight this serious disease, the government should take more stringent measures to significantly limit travel instead of simply advising citizens to stay at home.
